# MicroRNAs in the Pathogenesis, Diagnosis, Prognosis and Targeted Treatment of Cutaneous T-Cell Lymphomas

**DOI:** 10.3390/cancers12051229

**Published:** 2020-05-13

**Authors:** Maria Gluud, Andreas Willerslev-Olsen, Lise Mette Rahbek Gjerdrum, Lise M. Lindahl, Terkild B. Buus, Mads Hald Andersen, Charlotte Menne Bonefeld, Thorbjorn Krejsgaard, Ivan V. Litvinov, Lars Iversen, Jürgen C. Becker, Jenny L. Persson, Sergei B. Koralov, Thomas Litman, Carsten Geisler, Anders Woetmann, Niels Odum

**Affiliations:** 1 LEO Foundation Skin Immunology Research Center, Department of Immunology and Microbiology, University of Copenhagen, DK-2200 Copenhagen, Denmark; mgluud@sund.ku.dk (M.G.); awo@sund.ku.dk (A.W.-O.); terkild.buus@sund.ku.dk (T.B.B.); cmenne@sund.ku.dk (C.M.B.); thorkr@sund.ku.dk (T.K.); tlitman@sund.ku.dk (T.L.); cge@sund.ku.dk (C.G.); awoetmann@sund.ku.dk (A.W.); 2 Department of Pathology, Zealand University Hospital, DK-4000 Roskilde, Denmark; lmgj@regionsjaelland.dk; 3 Department of Clinical Medicine, University of Copenhagen, DK-2200 Copenhagen, Denmark; 4 Department of Dermatology, Aarhus University Hospital, DK-8200 Aarhus, Denmark; lise.lindahl@clin.au.dk (L.M.L.); lars.iversen@clin.au.dk (L.I.); 5 Center for Cancer Immune Therapy (CCIT), Department of Hematology and Oncology, Copenhagen University Hospital, Herlev Hospital, DK-2730 Herlev, Denmark; mads.hald.andersen@regionh.dk; 6 Division of Dermatology, McGill University Health Centre, Montreal, QC H4A 3J1, Canada; ivan.litvinov@mcgill.ca; 7 Translational Skin Cancer Research, German Cancer Consortium (DKTK), University Hospital Essen and Deutsches Krebsforschungszentrum (DKFZ), D-45141 Essen, Germany; j.becker@dkfz-heidelberg.de; 8 Department of Molecular Biology, Umea University, 90187 Umea, Sweden; jenny.persson@umu.se; 9 Department of Pathology, New York University School of Medicine, New York, NY 10016, USA; Sergei.Koralov@nyulangone.org

**Keywords:** microRNA, miR, cutaneous T-cell lymphoma, CTCL, mycosis fungoides, cancer, tumor suppressor, oncogene, biomarker, targeted therapy

## Abstract

Cutaneous T-cell lymphoma (CTCL) represents a heterogeneous group of potentially devastating primary skin malignancies. Despite decades of intense research efforts, the pathogenesis is still not fully understood. In the early stages, both clinical and histopathological diagnosis is often difficult due to the ability of CTCL to masquerade as benign skin inflammatory dermatoses. Due to a lack of reliable biomarkers, it is also difficult to predict which patients will respond to therapy or progress towards severe recalcitrant disease. In this review, we discuss recent discoveries concerning dysregulated microRNA (miR) expression and putative pathological roles of oncogenic and tumor suppressive miRs in CTCL. We also focus on the interplay between miRs, histone deacetylase inhibitors, and oncogenic signaling pathways in malignant T cells as well as the impact of miRs in shaping the inflammatory tumor microenvironment. We highlight the potential use of miRs as diagnostic and prognostic markers, as well as their potential as therapeutic targets. Finally, we propose that the combined use of miR-modulating compounds with epigenetic drugs may provide a novel avenue for boosting the clinical efficacy of existing anti-cancer therapies in CTCL.

## 1. Introduction

### 1.1. Etiology of Cutaneous T Cell Lymphoma

**Figure 1:**
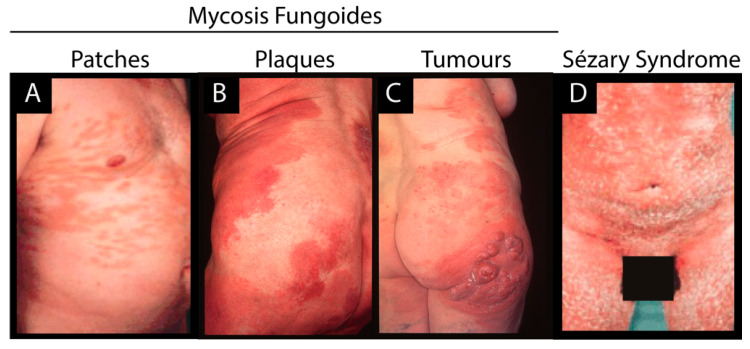
Stages and subtypes of cutaneous T-cell lymphoma (CTCL). (**A**), (**B**) and (**C**): The three main stages of mycosis fungoides (MF). (**D**) A patient suffering from Sézary syndrome (SS).

Cutaneous T-cell lymphoma (CTCL) comprises a heterogeneous group of non-Hodgkin lymphomas characterized by the proliferation of neoplastic T cells in a chronic inflammatory environment. CTCL can be divided into subgroups, with mycosis fungoides (MF) being the most prevalent form, and in most cases a relatively indolent cancer (Figure 1). Sézary syndrome (SS) is a rare, highly aggressive leukemic variant of CTCL and is associated with extensive exfoliative erythroderma (Figure 1) [1]. Early stage MF presents with patches and plaques and some patients may progress to advanced stages, presenting with cutaneous tumors and/or malignant T cell dissemination to lymph nodes, blood and visceral organs (Figure 1). MF lesions are frequently large, multifocal and highly pruritic; however, a broad range of MF variants have been described (e.g., folliculotropic, pagetoid reticulosis, hyperpigmented, granulomatous, granulomatous slack skin, poikiloderma vasculare atrophicans, unilesional and erythrodermic MF) [1,2]. Early diagnosis of CTCL (in particular MF) is difficult due to resemblance of CTCL with benign skin inflammatory dermatoses. Indeed, it may take up to 5 years and multiple skin biopsies to obtain a definitive diagnosis [1,2]. Moreover, it is generally impossible to predict which patients will suffer from an aggressive clinical course [3]. Despite intense research efforts in recent years, the pathogenesis of CTCL still remains poorly understood. Genetic, epigenetic, and environmental factors have all been proposed to be implicated in CTCL [1,4,5]. To date, no studies have identified overarching mutational drivers explaining the pathogenesis of this cancer; however, genetic aberrations may be important in a subset of patients, but are not sufficient to explain the full complexity of CTCL’s pathology [6]. Abnormalities of certain molecular pathways are frequently observed in CTCL and are believed to play an important role in a subset of patients [7]. Recurrent mutations include genetic abnormalities that facilitate constitutive activation of JAK/STAT signaling as well as oncogenic mutations that potentiate constitutive NFĸB signaling [8,9,10,11,12,13]. Recently, several lines of evidence have pointed towards a key role for epigenetic dysregulation in the pathogenesis of CTCL [14,15,16]. This is a notion further highlighted by the efficacy of therapeutic drugs targeting the genome’s epigenetic landscape (e.g., histone deacetylase (HDAC) inhibitors romidepsin and vorinostat) [17,18]. 

### 1.2. MicroRNAs in Health and Disease 

Non-coding RNA (ncRNA) molecules constitute a group of RNA transcripts that encompass microRNA (miR), long non-coding RNA (lncRNA) and circular RNA (circRNA) molecules. miRs are small, endogenous single-stranded 20–25 nucleotide RNA molecules and are one of the best characterized groups of ncRNAs [19]. miR biogenesis initiates with the transcription of miR genes, after which the primary miR transcripts are cleaved by enzymes including Drosha and then further processed by the ribonuclease Dicer to produce the mature and functioning miR form [20]. miRs have emerged as key post-transcriptional regulators, causing translational inhibition or degradation of mRNA by binding to the 3′ untranslated region of their target-mRNA, thereby fine-tuning protein expression [21]. miRs regulate at least 30% of human genes and play key roles in various aspects of normal cellular processes, including cell proliferation, differentiation, apoptosis and immune responses [19,21]. The role of dysregulated miRs has been elucidated in numerous types of inflammatory skin disorders and cancers. Notably, half of the miR-encoding loci are tumor-associated or located in fragile loci and were proposed to have modulatory effects on tumorigenesis [19,22]. Some miRs act as oncogenic miRs (oncomiRs), driving mechanisms that contribute to carcinogenesis, whereas others act as tumor suppressors. Interestingly, some miRs can have a dual role, depending on the context and the tissue where they are being expressed [19]. 

A large number of findings highlight the importance of investigating the role of aberrantly expressed miRs in CTCL pathogenesis and exploring their use in disease diagnosis and prognosis. 

## 2. Differentially Expressed miRs in CTCL

miR profiles have been extensively studied, and a large number of studies have reported on dysregulated miR expression in CTCL. miR-155 was among the first to be identified as being aberrantly overexpressed in this cancer and numerous studies have since highlighted miR-155 as a key player in oncogenesis [23,24,25,26,27]. Hundreds of miRs have been reported to be differentially expressed in patients diagnosed with tumor stage MF compared to controls. These include the upregulation of miR-93, miR-155, miR-181, miR-142 and downregulation of miR-200c, miR-203 and miR-205 [24,26,28]. In SS cohorts, miR-21, miR-181 and miR-214 were identified as highly expressed miRs, whereas miR-150 was shown to be downregulated [29,30,31,32]. Striking differences as well as similarities in miR patterns have been reported between CTCL variants [25,27,33]. Of particular interest, tumor stage MF and SS showed only few overlaps among differentially expressed miRs. Surprisingly, some miRs, such as miR-93 and members of the miR-17/92 cluster, were upregulated in tumor stage MF, but reduced in SS [27,31]. Likewise, a study comparing miR profiles in erythrodermic MF and SS (that are often clinically and histopathologically difficult to distinguish) demonstrated that erythrodermic MF is characterized by a miR expression profile resembling that of early MF rather than that of SS, thus underscoring that these are two distinct disease entities [33].

## 3. Dysregulation of miRs in CTCL

Dysregulated miRs are likely involved in disrupting normal cellular processes related to carcinogenesis in CTCL. The putative mechanisms leading to the dysregulation of miRs in CTCL have not been fully elucidated and several mechanisms may promote and/or inhibit the expression of miRs. Aberrant DNA methylation patterns of miR promoters were reported in CTCL [34]. Accordingly, miR-200c appears to be epigenetically silenced by promoter methylation in CTCL, contributing to enhanced activation of Notch signaling, associated with CTCL carcinogenesis [35,36]. Genomic analysis has revealed that DNA copy number variations in miR encoding genes may also contribute to aberrant expression of some miRs including high expression of miR-93, which facilitate sustained proliferation [27,37,38]. Furthermore, there are strong indications that abnormal regulation of transcription factors, including the constitutive activation of STAT signaling, drives the enhanced expression of miRs e.g., miR-155 and miR-21. These miRs are believed to promote oncogenesis through direct effects on proliferation/apoptosis in malignant T cells and indirectly by modulating the tumor microenvironment [39,40,41]. Simultaneously, aberrant STAT activation also seems to repress expression of tumor suppressor miRs, e.g., miR-22, further contributing to oncogenesis [42]. Moreover, dysregulation of the enzymes Dicer and Drosha, involved in miR biogenesis and processing, was reported in several cancers, resulting in defective processing and thus altered miR profiles in cancers [20]. Interestingly, increased expression of Dicer and reduced expression of Drosha was observed in CTCL, reflecting the complexity of miR dysregulation, which remains to be further understood [43,44].

A comprehensive study, taking advantage of the Danish cohort of twins, suggested that CTCL has very few heritable components, and several lines of evidence indicate that environmental factors may play a promoting or even initiating role in the carcinogenesis of CTCL [5,45]. Accordingly, it may be speculated that exogeneous factors including drugs, pesticides, and bacterial toxins may promote disease progression [5]. Indeed, in a recent study, Lindahl et al. [46] reported that aggressive antibiotic therapy inhibited skin colonization by toxin-producing Staphylococcus aureus (*Staph A*), decreased disease activity as well as the fraction of malignant T cells present in lesional skin in patients with advanced CTCL. These results suggest that bacteria may fuel disease activity [46]. Likewise, *Staph A*-derived toxins appear to tilt the balance between malignant and non-malignant infiltrative CD4+ T cells in the favor of enhanced survival of malignant T cells [47]. In support of a microbial driver of CTCL activity, Fanok et al. [48] demonstrated in a murine model of CTCL that disease progression is dependent on microbiota [48]. Of note, Willerslev-Olsen et al. documented that *Staph A*’s enterotoxins induce STAT5-mediated expression in malignant T cells obtained from SS patients [49]. As STAT5 activation was previously shown to trigger miR-155 expression and *Staph A*-derived enterotoxins induce miR-155 in T cells, it is likely that *Staph A* and its toxin trigger miR-155 in CTCL skin lesion in vivo [40,50]. Interestingly, *Helicobacter pylori* (*H. pylori*) and its cytotoxin-associated gene A (CagA) were shown to promote malignant transformation of gastic mucosal epithelial cells. *H. pylori* impairs DNA mismatch repair and facilitates tumor growth through the induction of miR-155, suggesting that *H. pylori,* at least partly, mediates carcinogenesis through miR-155 expression in gastric cancer [51,52]. Additionally, *H. pylori* has been reported to promote carcinogenesis via miR-155 upregulation in a model of gastric mucosa-associated lymphoid tissue (MALT) lymphoma [53]. Further studies are warranted to address the putative link between environmental factors such as *Staph A*, deregulated miR expression, and CTCL disease activity.

## 4. OncomiRs in CTCL Pathogenesis

Emerging evidence indicates that miRs are involved in the pathogenesis of CTCL. miR-155, miR-214 and miR-21 are to date the best established oncomiRs that drive carcinogenesis. 

### 4.1. Role of miR-21 in Malignant Proliferation

miR-21 is abundantly expressed in various tumors. Notably, it is upregulated in malignant T cells both in the tumor microenvironment of MF and in circulating cells isolated from SS patients [29,34,54]. Through chromatin immunoprecipitation (ChIP) analysis, it was found that elevated miR-21 expression is driven directly by transcription factors STAT3 and STAT5 [39,41]. Thus, activating mutations of these or cytokine-induced activation of STAT signaling promote high expression of miR-21. Several studies on SS patients indicated that abundant expression of miR-21 contributes to apoptotic resistance in CTCL [29]. Accordingly, miR-21 was able to promote cell survival in SS cells, and silencing of miR-21 in malignant T cells induced apoptosis [41]. The status of PTEN, a well-established tumor suppressor, is frequently downregulated in neoplastic cells, and miRs including miR-21 and miR-106b were suggested to play a role in reducing the expression of PTEN [55,56]. 

### 4.2. Role of miR-155 in Disease Progression 

The role of miR-155 is well-established in oncology, and the consequences of aberrant expression of miR-155 have also been addressed in relation to CTCL. Several reports indicate that miR-155 is important in promoting proliferation and tumor progression. Kopp et al. provided the first evidence that both malignant T cells and non-malignant T cells express miR-155 in CTCL lesions in situ [57]. Additionally, miR-155 was shown to be significantly upregulated in lymphoma cells in tumor stages compared to lymphocytes in early stages [58]. Functionally, miR-155 targets the global chromatin organizer and transcription factor, *Special AT-rich sequence-Binding protein 1 (SATB1)* [59]. Thus, *SATB1* inhibition by miR-155 in malignant T cells promotes proliferation and induces the expression of the Th2 cytokines IL-5 and IL-9, which are involved in CTCL pathogenesis as growth factors and inflammatory mediators [59,60]. The oncogenic role of miR-155 in CTCL is further supported by findings in an xenograft mouse model of CTCL, where treatment with a miR-155 inhibitor triggered enhanced apoptosis in malignant T cells [61]. 

Notably, in relation to STAT signaling in CTCL, it should be noted that aberrant STAT5 activation enhances the expression of the miR-155 host gene *BIC* (B-cell integration cluster) and miR-155, facilitating proliferation in malignant T cells [40]. In contrast, reports have revealed that the transcription factor STAT4, critical for Th1 phenotype differentiation, is downregulated in CTCL [62]. Loss of STAT4 is associated with the switch towards a Th2 inflammatory environment, subsequently orchestrating a tumor-promoting inflammatory state [63]. Interestingly, siRNA-mediated miR-155 knockdown enhanced STAT4 expression in malignant T cells, indicating that deficient STAT4 expression is, at least partly, driven by miR-155 [63]. Thus, miR-155 may also play a key role in the switch from Th1- to the Th2-dominant environment frequently observed in MF skin lesions during disease progression [62]. 

**Figure 2:**
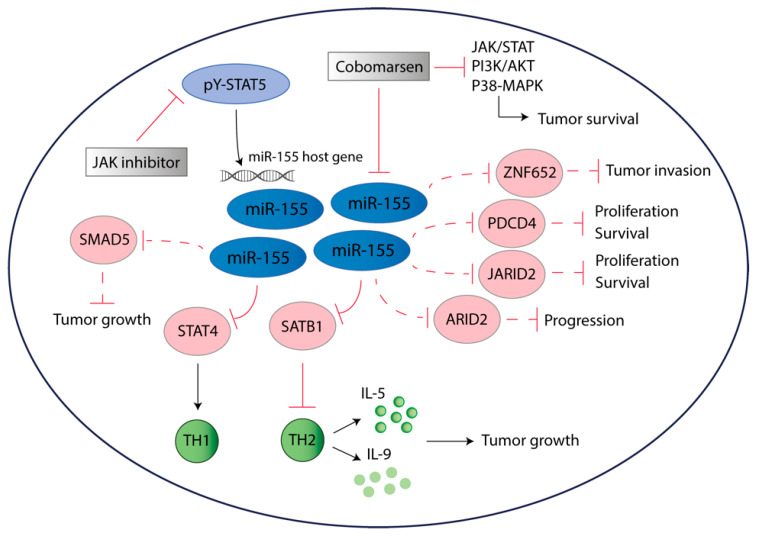
miR-155 promotes tumorigenesis in CTCL. Constitutive activation of STAT5 induces transcription and JAK inhibition represses the expression of miR-155. The oncomiR-155 exerts its functions through multiple pathways. It plays a role in switching the tumor microenvironment from Th1 to Th2 favoring by inhibition of *STAT4* and *SATB1*. Moreover, miR-155 may target several tumor suppressors including *JARID2, PDCD4, ZNF652, SMAD5* and *ARID2* (dashed lines), thus facilitating enhanced proliferation, decreased apoptosis, sustained survival and allowing tumor invasion. Targeting of miR-155 using Cobomarsen (currently being evaluated in phase 2 trials) decreases activity of several survival pathways including JAK/STAT, PI3K-AKT and p38-MAPK.

In addition to repressing SATB1 and STAT4 in CTCL, miR-155 regulates multiple signaling pathways of potential importance in malignant transformation. For instance, miR-155 targets several genes encoding tumor suppressors and inducers of apoptosis in other cancers (Table 1) [64]. To address whether miR-155 also represses these tumor suppressors in CTCL, we treated malignant T cells with anti-miR-155 and a non-targeting control prior to the analysis of changes in mRNA expression as previously described [59]. Interestingly, a series of well-established miR-155 targets such as *PDCD4, JARID2, ARID2, ZNF652*, and *SMAD5* displayed a ≥2-fold upregulation in malignant T cells following miR-155 inhibition (Table 1, right column, unpublished data). Thus, miR-155 may promote malignant transformation and disease progression of CTCL by the inhibition of multiple tumor suppressors and pro-apoptotic pathways in CTCL (Figure 2). Moreover, the literature indicates that miR-155 has several direct and indirect downstream targets that affect essential survival pathways such as JAK/STAT, PI3K-AKT, p38-MAPK [65].

### 4.3. Role of miR-214 in Promoting Cell Survival in SS

The human miR-214 gene is located in an intron of the *DNM3* gene. DNM3 is known to be overexpressed in SS and the gene is regulated by SS-associated transcription factors including *TWIST1*, potentially accounting for the abundant expression of miR-214 in SS [29,30,82]. In contrast to miR-21, miR-214 is predominantly overexpressed in circulating malignant T cells [82]. Addressing the functional role of miR-214 revealed that miR-214 promotes cell survival in malignant SS cells, thus facilitating apoptosis resistance in CTCL [29]. Furthermore, the inhibition of miR-214 was shown to decrease disease severity in a CTCL disease mouse model [83]. 

### 4.4. The miR-17/92 Cluster—Role as oncomiRs or Tumor Suppressor miRs in CTCL?

Members of the miR cluster miR-17/92 have been proposed to act as both tumor suppressors and oncomiRs in cancers depending on the cellular context [84]. In CTCL, several studies have addressed the expression of this miR cluster. Downregulation of the miR-17/92 cluster was observed in SS T cells compared to healthy controls. Moreover, ectopic overexpression of miR-17-5p in SS T cells improved the proliferative ability concomitant with increased apoptosis in these cells [31]. Notably, another study reported that the expression of miR-17-3p was increased in SS T cells compared to healthy controls [29]. In advanced MF lesions, the expression of this cluster was found to be upregulated compared to early MF stages or benign inflammatory dermatoses [27,54]. Interestingly, different levels of the miR cluster were associated with different types of MF. Abundant expression of the miR-17/92 members was reported in unilesional MF, exhibiting a less aggressive type compared to MF multifocal early disease. Furthermore, increased levels of the miR-17/92 cluster members correlated with a Th1-skewed cytokine microenvironment [85]. Thus, the expression of miR-17/92 was suggested to contribute to a robust reactive T cell immune response, likely to explain the less aggressive disease phenotype [85]. Taken together, discrepancies regarding the role of the miR-17/92 cluster exist in CTCL, and differences in miR expression levels in different cell types and cellular settings may explain these inconsistencies. 

## 5. Key Tumor Suppressive miRs Playing a Role in CTCL Pathogenesis

Overcoming apoptosis and senescence while sustaining proliferation and promoting invasion are hallmarks of cancer [86]. In addition to driving tumorigenesis, miRs may also exhibit tumor-suppressive properties, and the repression of such miRs may result in the progression of cancer. Studies identifying miRs as tumor suppressors in CTCL have revealed that miR-337 is substantially downregulated in malignant T cell lines suggestive of its tumor suppressive role. Indeed, ectopic overexpression of miR-337 suppressed key aspects of CTCL such as viability and invasion. The tumor-suppressive properties were induced through direct repression of STAT3 activity, essential for CTCL tumorigenesis [11,87]. Other findings suggest that miR-150 is an important player in CTCL tumor invasion. miR-150 shapes the inflammatory tumor microenvironment of advanced CTCL by repressing the autocrine IL-22-CCL20-CCR6 signaling [88]. Consequently, diminished expression of miR-150 induces CCR6 chemotaxis, subsequently facilitating the migrative potential of CTCL cells [88]. Furthermore, miR-16 has been shown to be significantly downregulated in advanced disease [89]. Notably, forced expression of miR-16 induced expression of the cell cycle regulator p21, leading to cell cycle arrest [89,90].

In addition, other miRs may play a role in CTCL, including miR-22, miR-135a and miR-223. Diminished expression of miR-135a, miR-223 and miR-22 may allow for the high expression of critical transcription factors including GATA3 as well as growth and proto-oncogenes such as E2F1 and c-Myc, respectively, collectively contributing to CTCL pathogenesis and progression [42,91,92]. 

## 6. Non-Canonical Functions of miRs

Chronic itch is a major symptom in patients suffering from CTCL. For years, it remained unknown which mediators may be involved in causing it. Some lines of evidence suggested IL-31 as an important driver of pruritus. However, this notion is still controversial, as other studies did not observe an association between Il-31 and severe itch in CTCL patients [93,94,95]. Recently, Han et al. demonstrated that miR-711, which is highly expressed in CTCL, promotes itching through an unconventional signaling mechanism, as miR-711 was shown to evoke itching by binding extracellularly to the ion channel TRPA1 on sensory neurons [96]. 

Additionally, miRs can act as physiological ligands for specific Toll like receptors (TLRs) and initiate signaling cascades of immune responses [97,98]. Interestingly, miRs known to be upregulated in CTCL, such as miR-21 and several let-7 family members, have been demonstrated to serve as ligands for TLRs in different cells types, modulating complex mechanistic networks [54,98,99]. Thus, unconventional miR signaling may be of future therapeutic interest for CTCL.

An overview of the miRs known to play a role in CTCL pathogenesis is summarized in Table 2. 

## 7. miRs as Diagnostic, Prognostic and Treatment Predictive Biomarkers

In clinical practice, MF and SS remain challenging to diagnose and distinguish from benign inflammatory skin disorders [1]. The median time from initial appearance of skin lesions to the diagnosis of MF cases is around 4 years [1]. Notably, delays in diagnosis have been found to exceed four decades in some patients [1]. As discussed above, there is increasing evidence that skin diseases and certain cancers demonstrate unique miR profiles. Thus, aberrant miR expression signatures may help to diagnose and prognosticate CTCL. To date, a number of clinical trials are actively testing the value of using miRs as biomarkers or have already been completed [100]. 

Early MF disease often follows an indolent course, but some patients progress to more advanced stages rapidly. It would be an advantage in MF management to be able to predict patients at high risk of developing the aggressive disease [1,3]. Furthermore, it could be hypothesized that earlier initiation of systemic treatments and enhanced clinical monitoring may increase overall life expectancy in these patients. This could also apply to patients diagnosed with the aggressive CTCL variant SS, that in general has poor prognosis, and where early diagnosis potentially would be important to achieve enhanced therapeutic response. 

In 2011, the first diagnostic miR profile able to differentiate between MF and benign skin inflammatory disorders was published. The profile includes a decreased expression of miR-203 and miR-205 and increased expression of miR-155 [101]. The study was confirmed in an independent cohort of patients [102], and subsequently, Ralfkiaer et al. reported that early MF and atopic dermatitis display very different miR patterns, including different levels of miR-203, miR-205 and miR-155 [54]. Accordingly, a long list of dysregulated miRs were identified, including miR-181 and miR-146a [54]. Numerous miRs were further identified as being associated with a progressive disease. Notably, miRs such as miR-93, miR-106b and miR-181 are found to be associated with advanced stages of MF, suggesting a possible role in malignant transformation and progression [54]. Other investigations have suggested that a minimal classifier of just four miRs (miR-181a, miR-146a, miR-222, miR-26a) could discriminate tumor stage MF from benign conditions [28]. Interestingly, two of these miRs (miR-181a, miR-146a) were proposed to be involved in the progression from early MF to advanced disease (stage >IIB) [54,103]. Of notice, a recent study examining a cohort of Chinese CTCL patients validated the relevance of the aforementioned diagnostic classifiers [28]. Inclusion of miR-130b, miR-142-3p, miR-200c appeared to further strengthen the diagnostic potential. However, miR-205 did not contribute to the diagnostic strengths and accuracy of the classifier in this study, potentially due to ethnic differences in the cohorts. This highlights the importance of validating miR classifiers in cohorts of different ethnic backgrounds [28]. The discovery of circulating miRs in human plasma sparked intensive research, as these could serve as accessible and hence convenient biomarkers. miRs may be detected in the blood as a result of the passive leakage of cells including tumor cells, or they can be present in cell-secreted exosomes. Circulating miRs in the blood are very stable and not easily degraded, thus possessing the ability to be applied for clinical monitoring [104]. In CTCL, the 3-miR classifier (miR-203, miR-205, miR-155) was evaluated in a study investigating plasma levels of miRs and proposed that screening plasma levels for these molecules could be a valuable tool for diagnosis and monitoring the disease activity and progression in CTCL [104]. 

In addition to using miR profiling as a diagnostic tool in CTCL, miR profiling also emerged as a potentially important prognostic tool. This is of key clinical importance, as it is notoriously difficult to predict which patients are at risk of disease progression and, therefore, are in need of special attention and aggressive therapy. This is supported by the fact that non-progressive disease usually takes an indolent course with a good prognosis and an almost normal life expectancy, while patients with progressive disease often only display partial or transient responses to treatments and have a poor prognosis [1]. To investigate the potential use of miR profiling for disease prognostication, Lindahl et al. [3] investigated a large cohort of early MF patients with the goal to identify and validate a prognostic miR classifier. This miR classifier, consisting of miR-106b-5p, miR-148a-3p and miR-338-3p, successfully predicted patients at high risk of a progressive disease course and with reduced overall survival [3].

Moreover, miR profiles may also predict the response to therapy. Specifically, a recent study reported that MF patients who had a rapid increase in miR-223, miR-191 and miR-342 levels following extracorporeal photopheresis were more likely to demonstrate a favorable clinical response after 6–12 months [105]. Furthermore, a number of reports demonstrated that miRs may determine the response to cancer therapy, which is often hampered by therapeutic resistance and presents a major clinical challenge. For instance, the c-Myc/mirR-125b signaling pathway was shown to regulate the sensitivity of tumor cells toward the proteasome inhibitor bortezomib in a pre-clinical model of CTCL. According to the study, high expression of miR-125b increased cellular resistance and tumor growth [106]. Moreover, upregulation of another miR, miR-122, was shown to act as an amplifier in an antiapoptotic signaling circuit, decreasing the sensitivity in CTCL to chemotherapy [107]. A predictive biomarker may not only reduce valuable time spent on futile and toxic therapies but may also help in tailoring the optimal treatment for individual patients.

**Figure 3:**
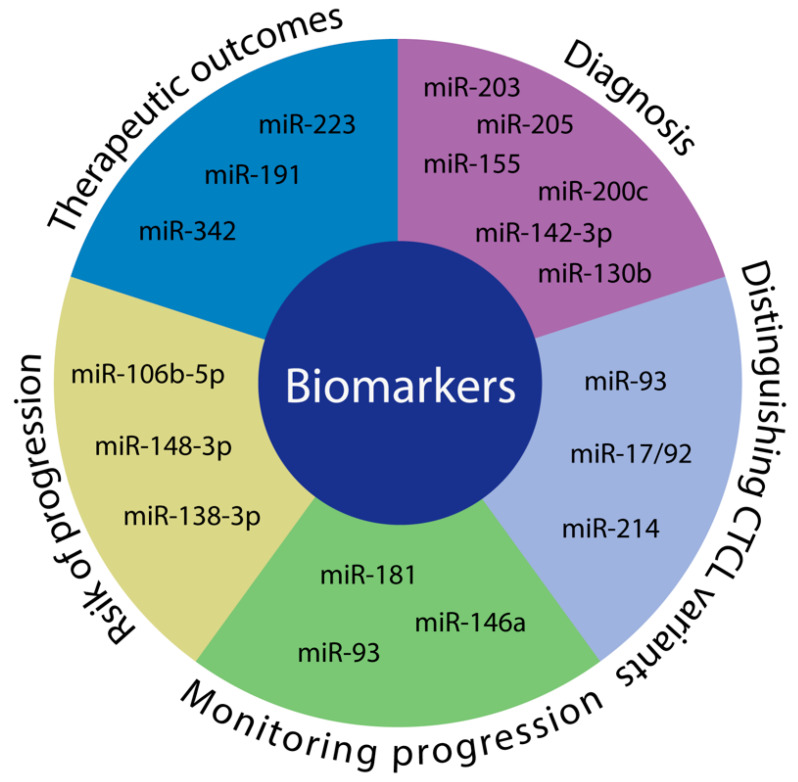
miRs as diagnostic, prognostic and predictive biomarkers. miRs possess an important clinical potential to be used as biomarkers. In CTCL, evidence suggests that they may be able to discern CTCL from benign inflammatory dermatoses (diagnosis) as well as distinguish between subtypes of CTCL (distinguishing CTCL variants). Furthermore, miRs may be able to stratify patients into low vs. high risk of progression groups (risk of progression) and could be used to monitor progression in patients (monitoring progression). Additionally, expression of certain miRs may be able to predict treatment outcomes (therapeutic outcomes).

Taken together, emerging evidence suggests an important clinical potential of miRs as biomarkers in CTCL (Figure 3). Prognostic and treatment predictive miR profiles may pave the way for implementation of personalized medicine in these patients. Several of the miRs that were reported to play a key role as biomarkers in CTCL also have a functional impact in CTCL pathology, therefore potentially serving as relevant targets for future therapies. Larger studies are still highly warranted to fully establish miRs as diagnostic, prognostic and predictive classifiers in CTCL.

## 8. Dual Interplay between miRs and Therapeutics Targeting the Epigenome

Alterations in the epigenome are known to be present in a variety of diseases, and emerging insights suggest that epigenetic modifiers are promising targets in cancer therapy [1]. HDAC inhibitors are clinically approved for the treatment of CTCL and have already been proven effective in everyday clinical practice [1,17]. Several reports have investigated the role of HDAC inhibitors in modulating the miR environment in CTCL. Importantly, the HDAC inhibitor vorinostat restored epigenetic silencing of tumor suppressive miRs including miR-16, miR-22 and miR-150 leading to increased apoptosis in CTCL as well as attenuating the migrative potential of CTCL cells [42,89,108]. Epigenetic regulation also seems to be of pivotal importance in reverting the high expression of miRs, including miR-93, as its expression is reduced by vorinostat treatment [38].

Excessive expression of cytokines including IL-15 has been implicated in CTCL pathogenesis [109]. Autocrine IL-15 signaling drives induction of several HDACs along with an increased expression of oncomiR-21 [110]. Moreover, IL-15 has been shown to promote reduction in the expression level of miR-29b, which is involved in regulating bromodomain-containing protein 4 (BRD4), an epigenetic reader protein that contributes to the modulation of the epigenetic landscape by recognizing and binding to epigenetic modifications [111]. The oncogenic activity of BRD4 was inhibited by restoring the miR-29b activity by bortezomib treatment or directly by a bromodomain inhibitor (JQ1), both interventions leading to the prevention of disease progression in CTCL [111].

## 9. miR Targeted Therapy

Based on the discussed literature, it is evident that miR-targeting drugs possess the potential to restore homeostasis of multiple oncogenic networks in CTCL. Indeed, preclinical validations of numerous miR drug candidates have been reported and early active phase trials of miR are currently being performed; however, phase 3 trials targeting miRs still remain to be initiated [83,100,112].

As a consequence of the ever expanding body of evidence highlighting the implication of miR-155 in CTCL pathogenesis, cobomarsen (MRG-106), an oligonucleotide inhibitor of miR-155 was recently developed [65]. It is anticipated that the inhibition of miR-155 will reverse the malignant phenotype of malignant T cells, preventing the oncogene network from facilitating a constitutive survival loop in CTCL. In a preclinical study, it was observed that cobomarsen inhibits cell proliferation and induces apoptosis in CTCL cells. Moreover, cobomarsen reduces the activation of oncogenic signaling pathways such as the JAK/STAT and PI3K/AKT (Figure 2) [65]. Its proliferative inhibition was comparable to the effects observed by treating with bexarotene, a commonly prescribed rexinoid medication in CTCL [65]. Phase 1 trials were encouraging as they reported cobomarsen to be well tolerated with signs of a favorable clinical activity [112,113]. Studies are currently recruiting patients for phase 2 clinical trials in CTCL [112,113]. Given the dual interplay between miR-155 and JAK/STAT with STAT5 inducing transcription of the miR-155 host gene and miR-155 promoting JAK/STAT signaling, it is tempting to speculate that combined targeting of both miR-155 and JAK/STAT5 may act in synergism. In support, JAK/STAT is believed to also promote oncogenesis in a miR-155-independent manner [42,114,115]. On the other hand, miR-155 expression can also be induced in a STAT-independent fashion relying on other transcription factors, potentially NFĸB [65,116]. Other miR targeted therapies may also exhibit clinical utility for the treatment of CTCL. Of particular interest are inhibitors targeting miR-214 and miR-21 or miR mimics restoring tumor suppressor miRs. Preclinical studies demonstrated that antagomiR-214 treatment significantly decreased disease severity in a CTCL mouse model, and miR-21 seems to be highly involved in promoting malignant growth [83]. Thus, continued efforts to uncover which miRs possess potential as therapeutic targets are still warranted [100].

## 10. Conclusions

Accumulating evidence suggests that aberrant expression of numerous miRs participates in driving oncogenic survival pathways and disease progression in CTCL. Efforts to further elucidate and validate the diagnostic, prognostic and predictive miR classifiers may pave the way for both earlier diagnosis, improved monitoring, risk-assessment of disease progression, predicting treatment outcomes and eventually enabling us to implement personalized medicine. Moreover, miR targeting therapies alone or in combination with epigenetic drugs may provide a novel avenue for improving the clinical efficacy of treatments for CTCL patients.

## Figures and Tables

**Table 1:** Putative miR-155 targets in CTCL.

miR-155 Targets	Role in Cancers	Validated Functions	Fold Change	References
*JARID2*	Tumor suppressor in leukemia	Reduces proliferation and survival	2.2	[66,67,68,69]
*PDCD4*	Tumor suppressor in leukemia, tongue-, colorectal- and cervical cancer, mutated in SS	Increases apoptosis Induces cell cycle arrest	2.0	[70,71,72,73,74]
*ZNF652*	Tumor suppressor in breast cancer	Represses drivers of invasion and metastasis	3.4	[75,76]
*SMAD5*	Tumor suppressor in leukemia and B-cell lymphoma	Plays a role in growth inhibition of malignant cells	2.2	[77,78,79]
*ARID2*	Tumor suppressor in hepatocellular carcinoma and oral cancer, mutated in SS	Suppresses growth and progression of tumor cells	2.1	[64,80,81]

Table 1 The malignant T cell line MF2059, established from an MF patient, was treated with anti-miR-155 and a non-targeting control prior to the analysis of changes in mRNA expression, as previously described [59].

**Table 2:** Roles of miRs in CTCL pathogenesis.

MicroRNA	Status in CTCL	Validated Functions
*miR-16*	Downregulated in CTCL Upregulated by HDAC inhibition	Increases p21 and cell cycle arrest
*miR-17/92*	Upregulated in tumor MF Reduced in SS	Influences cytokines in MF Tumor suppressive properties in SS
*miR-21*	Upregulated in CTCL Driven by STAT signaling	Targets PTEN Increases cell survival
*miR-22*	Downregulated in CTCL Repressed by STAT signaling	Suppresses c-Myc
*miR-29b*	Downregulated in CTCL Reduced by IL-15	Suppresses BRD4
*miR-93*	Upregulated in tumor MF Reduced in SS	Represses p21 expression Induces proliferation in MF
*miR-122*	Upregulated in CTCL	Decreases sensitivity to chemotherapy
*miR-125b*	OncomiR in CTCL Regulated by c-Myc	Represses sensitivity to Bortezomib
*miR-135a*	Downregulated in CTCL	Suppresses GATA3
*miR-150*	Downregulated in advanced CTCL Upregulated by HDAC inhibition	Shapes the inflammatory environment Inhibits migration
*miR-155*	Upregulated in CTCL Driven by STAT signaling	Increases proliferation and survival Targets multiple pathways
*miR-200c*	Downregulated in CTCL Silenced by promotor methylation	Inhibits Notch signaling
*miR-214*	Upregulated in SS Driven by TWIST1 and BRD4	Promotes cell survival Increases disease severity in mouse models
*miR-223*	Downregulated in CTCL	Suppresses E2F1 Reduces cell growth
*miR-337*	Downregulated in CTCL	Targets STAT3 signaling Inhibits cell viability
*miR-711*	Upregulated in CTCL	Causes pruritus

## References

[1] Hristov A.C., Tejasvi T., Wilcox R.A. (2019). Mycosis fungoides and Sézary syndrome: 2019 update on diagnosis, risk-stratification, and management. Am. J. Hematol..

[2] Kim E.J., Hess S., Richardson S.K., Newton S., Showe L.C., Benoit B.M., Ubriani R., Vittorio C.C., Junkins-Hopkins J.M., Wysocka M. (2005). Immunopathogenesis and therapy of cutaneous T cell lymphoma. J. Clin. Investig..

[3] Lindahl L.M., Besenbacher S., Rittig A.H., Celis P., Willerslev-Olsen A., Gjerdrum L.M.R., Krejsgaard T., Johansen C., Litman T., Woetmann A. (2018). Prognostic miRNA classifier in early-stage mycosis fungoides: Development and validation in a Danish nationwide study. Blood.

[4] Litvinov I.V., Shtreis A., Kobayashi K., Glassman S., Tsang M., Woetmann A., Sasseville D., Ødum N., Duvic M. (2016). Investigating potential exogenous tumor initiating and promoting factors for Cutaneous T-Cell Lymphomas (CTCL), a rare skin malignancy. Oncoimmunology.

[5] Ghazawi F.M., Alghazawi N., Le M., Netchiporouk E., Glassman S.J., Sasseville D., Litvinov I.V. (2019). Environmental and Other Extrinsic Risk Factors Contributing to the Pathogenesis of Cutaneous T Cell Lymphoma (CTCL). Front. Oncol..

[6] Choi J., Goh G., Walradt T., Hong B.S., Bunick C.G., Chen K., Bjornson R.D., Maman Y., Wang T., Tordoff J. (2015). Genomic landscape of cutaneous T cell lymphoma. Nat. Genet..

[7] Netchiporouk E., Gantchev J., Tsang M., Thibault P., Watters A.K., Hughes J.-D.M., Ghazawi F.M., Woetmann A., Ødum N., Sasseville D. (2017). Analysis of CTCL cell lines reveals important differences between mycosis fungoides/Sézary syndrome vs. HTLV-1+ leukemic cell lines. Oncotarget.

[8] Ødum N. (2020). Deregulated signalling and inflammation in cutaneous T-cell lymphoma. Br. J. Dermatol..

[9] Kiel M.J., Sahasrabuddhe A.A., Rolland D.C.M., Velusamy T., Chung F., Schaller M., Bailey N.G., Betz B.L., Miranda R.N., Porcu P. (2015). Genomic analyses reveal recurrent mutations in epigenetic modifiers and the JAK–STAT pathway in Sézary syndrome. Nat. Commun..

[10] Yumeen S., Girardi M. (2020). Insights Into the Molecular and Cellular Underpinnings of Cutaneous T Cell Lymphoma. Yale J. Biol. Med..

[11] Seffens A., Herrera A., Tegla C., Buus T.B., Hymes K.B., Ødum N., Geskin L.J., Koralov S.B. (2019). STAT3 Dysregulation in Mature T and NK Cell Lymphomas. Cancers.

[12] Torres A.N.B., Cats D., Mei H., Szuhai K., Willemze R., Vermeer M.H., Tensen C.P. (2018). Genomic analysis reveals recurrent deletion of JAK-STAT signaling inhibitors HNRNPK and SOCS1 in mycosis fungoides. Geneschromosomes Cancer.

[13] Brender C., Nielsen M., Kaltoft K., Mikkelsen G., Zhang Q., Wasik M., Billestrup N., Odum N. (2001). STAT3-mediated constitutive expression of SOCS-3 in cutaneous T-cell lymphoma. Blood.

[14] Yi S., Sun J., Qiu L., Fu W., Wang A., Liu X., Yang Y., Kadin M.E., Tu P., Wang Y. (2018). Dual Role of EZH2 in Cutaneous Anaplastic Large Cell Lymphoma: Promoting Tumor Cell Survival and Regulating Tumor Microenvironment. J. Investig. Dermatol..

[15] Qiu L., Liu F., Yi S., Li X., Liu X., Xiao C., Lian C.G., Tu P., Wang Y. (2018). Loss of 5-Hydroxymethylcytosine Is an Epigenetic Biomarker in Cutaneous T-Cell Lymphoma. J. Investig. Dermatol..

[16] Li Y., Wang J., Yu M., Wang Y., Zhang H., Yin J., Li Z., Li T., Yan H., Li F. (2018). SNF5 deficiency induces apoptosis resistance by repressing SATB1 expression in Sézary syndrome. Leuk. Lymphoma.

[17] Lopez A.T., Bates S., Geskin L. (2018). Current Status of HDAC Inhibitors in Cutaneous T-cell Lymphoma. Am. J. Clin. Derm..

[18] Fantin V.R., Loboda A., Paweletz C.P., Hendrickson R.C., Pierce J.W., Roth J.A., Li L., Gooden F., Korenchuk S., Hou X.S. (2008). Constitutive activation of signal transducers and activators of transcription predicts vorinostat resistance in cutaneous T-cell lymphoma. Cancer Res..

[19] Si W., Shen J., Zheng H., Fan W. (2019). The role and mechanisms of action of microRNAs in cancer drug resistance. Clin. Epigenetics.

[20] Lin S., Gregory R.I. (2015). MicroRNA biogenesis pathways in cancer. Nat. Rev. Cancer.

[21] Ali Syeda Z., Langden S.S.S., Munkhzul C., Lee M., Song S.J. (2020). Regulatory Mechanism of MicroRNA Expression in Cancer. Int. J. Mol. Sci..

[22] Calin G.A., Sevignani C., Dumitru C.D., Hyslop T., Noch E., Yendamuri S., Shimizu M., Rattan S., Bullrich F., Negrini M. (2004). Human microRNA genes are frequently located at fragile sites and genomic regions involved in cancers. Proc. Natl. Acad. Sci. USA.

[23] Maj J., Jankowska-Konsur A., Sadakierska-Chudy A., Noga L., Reich A. (2012). Altered microRNA expression in mycosis fungoides. Br. J. Dermatol..

[24] Marosvári D., Téglási V., Csala I., Marschalkó M., Bödör C., Timár B., Csomor J., Hársing J., Reiniger L. (2015). Altered microRNA expression in folliculotropic and transformed mycosis fungoides. Pathol. Oncol. Res..

[25] Benner M.F., Ballabio E., van Kester M.S., Saunders N.J., Vermeer M.H., Willemze R., Lawrie C.H., Tensen C.P. (2012). Primary cutaneous anaplastic large cell lymphoma shows a distinct miRNA expression profile and reveals differences from tumor-stage mycosis fungoides. Exp. Dermatol..

[26] Papadavid E., Braoudaki M., Bourdakou M., Lykoudi A., Nikolaou V., Tounta G., Ekonomidi A., Athanasiadis E., Spyrou G., Antoniou C. (2016). Aberrant microRNA expression in tumor mycosis fungoides. Tumour Biol..

[27] Van Kester M.S., Ballabio E., Benner M.F., Chen X.H., Saunders N.J., van der Fits L., van Doorn R., Vermeer M.H., Willemze R., Tensen C.P. (2011). miRNA expression profiling of mycosis fungoides. Mol. Oncol..

[28] Shen X., Wang B., Li K., Wang L., Zhao X., Xue F., Shi R., Zheng J. (2018). MicroRNA Signatures in Diagnosis and Prognosis of Cutaneous T-Cell Lymphoma. J. Investig. Dermatol..

[29] Narducci M.G., Arcelli D., Picchio M.C., Lazzeri C., Pagani E., Sampogna F., Scala E., Fadda P., Cristofoletti C., Facchiano A. (2011). MicroRNA profiling reveals that miR-21, miR486 and miR-214 are upregulated and involved in cell survival in Sézary syndrome. Cell Death Dis..

[30] Qin Y., Buermans H.P.J., van Kester M.S., van der Fits L., Out-Luiting J.J., Osanto S., Willemze R., Vermeer M.H., Tensen C.P. (2012). Deep-Sequencing Analysis Reveals that the miR-199a2/214 Cluster within DNM3os Represents the Vast Majority of Aberrantly Expressed MicroRNAs in Sézary Syndrome. J. Investig. Dermatol..

[31] Ballabio E., Mitchell T., van Kester M.S., Taylor S., Dunlop H.M., Chi J., Tosi I., Vermeer M.H., Tramonti D., Saunders N.J. (2010). MicroRNA expression in Sezary syndrome: Identification, function, and diagnostic potential. Blood.

[32] Kohnken R., Kodigepalli K.M., Mishra A., Porcu P., Wu L. (2017). MicroRNA-181 contributes to downregulation of SAMHD1 expression in CD4+ T-cells derived from Sèzary syndrome patients. Leuk. Res..

[33] Rittig A.H., Lindahl L.M., Johansen C., Celis P., Ødum N., Iversen L., Litman T. (2019). The MicroRNA Expression Profile Differs Between Erythrodermic Mycosis Fungoides and Sézary Syndrome. Acta Derm. Venereol..

[34] Sandoval J., Díaz-Lagares A., Salgado R., Servitje O., Climent F., Ortiz-Romero P.L., Pérez-Ferriols A., Garcia-Muret M.P., Estrach T., Garcia M. (2015). MicroRNA expression profiling and DNA methylation signature for deregulated microRNA in cutaneous T-cell lymphoma. J. Investig. Dermatol..

[35] Gallardo F., Sandoval J., Díaz-Lagares A., Garcia R., D’Altri T., González J., Alegre V., Servitje O., Crujeiras A.-B., Stefánsson Ó.-A. (2015). Notch1 Pathway Activation Results from the Epigenetic Abrogation of Notch-Related MicroRNAs in Mycosis Fungoides. J. Investig. Dermatol..

[36] Kamstrup M.R., Gjerdrum L.M.R., Biskup E., Thyssing Lauenborg B., Ralfkiaer E., Woetmann A., Ødum N., Gniadecki R. (2010). Notch1 as a potential therapeutic target in cutaneous T-cell lymphoma. Blood.

[37] Garaicoa F.H., Roisman A., Arias M., Trila C., Fridmanis M., Abeldaño A., Vanzulli S., Narbaitz M., Slavutsky I. (2016). Genomic imbalances and microRNA transcriptional profiles in patients with mycosis fungoides. Tumour Biol..

[38] Gluud M., Fredholm S., Blümel E., Willerslev-Olsen A., Buus T.B., Nastasi C., Krejsgaard T., Bonefeld C.M., Woetmann A., Iversen L. (2020). MicroRNA-93 Targets p21 and Promotes Proliferation in Mycosis Fungoides T Cells. Dermatology.

[39] Lindahl L.M., Fredholm S., Joseph C., Nielsen B.S., Jønson L., Willerslev-Olsen A., Gluud M., Blümel E., Petersen D.L., Sibbesen N. (2016). STAT5 induces miR-21 expression in cutaneous T cell lymphoma. Oncotarget.

[40] Kopp K.L., Ralfkiaer U., Gjerdrum L.M.R., Helvad R., Pedersen I.H., Litman T., Jønson L., Hagedorn P.H., Krejsgaard T., Gniadecki R. (2013). STAT5-mediated expression of oncogenic miR-155 in cutaneous T-cell lymphoma. Cell Cycle.

[41] Van der Fits L., van Kester M.S., Qin Y., Out-Luiting J.J., Smit F., Zoutman W.H., Willemze R., Tensen C.P., Vermeer M.H. (2011). MicroRNA-21 expression in CD4+ T cells is regulated by STAT3 and is pathologically involved in Sézary syndrome. J. Investig. Dermatol..

[42] Sibbesen N.A., Kopp K.L., Litvinov I.V., Jønson L., Willerslev-Olsen A., Fredholm S., Petersen D.L., Nastasi C., Krejsgaard T., Lindahl L.M. (2015). Jak3, STAT3, and STAT5 inhibit expression of miR-22, a novel tumor suppressor microRNA, in cutaneous T-Cell lymphoma. Oncotarget.

[43] Valencak J., Schmid K., Trautinger F., Wallnöfer W., Muellauer L., Soleiman A., Knobler R., Haitel A., Pehamberger H., Raderer M. (2011). High expression of Dicer reveals a negative prognostic influence in certain subtypes of primary cutaneous T cell lymphomas. J. Dermatol. Sci..

[44] Gambichler T., Salveridou K., Schmitz L., Käfferlein H.U., Brüning T., Stockfleth E., Sand M., Lang K. (2019). Low Drosha protein expression in cutaneous T-cell lymphoma is associated with worse disease outcome. J. Eur. Acad. Derm. Venereol..

[45] Odum N., Lindahl L.M., Wod M., Krejsgaard T., Skytthe A., Woetmann A., Iversen L., Christensen K. (2017). Investigating heredity in cutaneous T-cell lymphoma in a unique cohort of Danish twins. Blood Cancer J..

[46] Lindahl L.M., Willerslev-Olsen A., Gjerdrum L.M.R., Nielsen P.R., Blümel E., Rittig A.H., Celis P., Herpers B., Becker J.C., Stausbøl-Grøn B. (2019). Antibiotics inhibit tumor and disease activity in cutaneous T cell lymphoma. Blood.

[47] Blümel E., Willerslev-Olsen A., Gluud M., Lindahl L.M., Fredholm S., Nastasi C., Krejsgaard T., Surewaard B.G.J., Koralov S.B., Hu T. (2019). Staphylococcal alpha-toxin tilts the balance between malignant and non-malignant CD4+ T cells in cutaneous T-cell lymphoma. OncoImmunology.

[48] Fanok M.H., Sun A., Fogli L.K., Narendran V., Eckstein M., Kannan K., Dolgalev I., Lazaris C., Heguy A., Laird M.E. (2018). Role of Dysregulated Cytokine Signaling and Bacterial Triggers in the Pathogenesis of Cutaneous T-Cell Lymphoma. J. Investig. Derm..

[49] Willerslev-Olsen A. Staphylococcus aureus enterotoxins induce FOXP3 in neoplastic T cells in Sézary syndrome. Blood Cancer J..

[50] Rao R., Rieder S.A., Nagarkatti P., Nagarkatti M. (2014). Staphylococcal enterotoxin B-induced microRNA-155 targets SOCS1 to promote acute inflammatory lung injury. Infect. Immun..

[51] Santos J.C., Brianti M.T., Almeida V.R., Ortega M.M., Fischer W., Haas R., Matheu A., Ribeiro M.L. (2017). Helicobacter pylori infection modulates the expression of miRNAs associated with DNA mismatch repair pathway. Mol. Carcinog..

[52] Ou Y., Ren H., Zhao R., Song L., Liu Z., Xu W., Liu Y., Wang S. (2019). Helicobacter pylori CagA promotes the malignant transformation of gastric mucosal epithelial cells through the dysregulation of the miR-155/KLF4 signaling pathway. Mol. Carcinog..

[53] Floch P., Capdevielle C., Staedel C., Izotte J., Sifré E., Laur A.M., Giese A., Korolik V., Dubus P., Mégraud F. (2017). Deregulation of MicroRNAs in Gastric Lymphomagenesis Induced in the d3Tx Mouse Model of Helicobacter pylori Infection. Front. Cell Infect. Microbiol..

[54] Ralfkiaer U., Lindahl L.M., Lindal L., Litman T., Gjerdrum L.-M., Ahler C.B., Gniadecki R., Marstrand T., Fredholm S., Iversen L. (2014). MicroRNA expression in early mycosis fungoides is distinctly different from atopic dermatitis and advanced cutaneous T-cell lymphoma. Anticancer Res..

[55] Cristofoletti C., Picchio M.C., Lazzeri C., Tocco V., Pagani E., Bresin A., Mancini B., Passarelli F., Facchiano A., Scala E. (2013). Comprehensive analysis of PTEN status in Sezary syndrome. Blood.

[56] Hollander M.C., Blumenthal G.M., Dennis P.A. (2011). PTEN loss in the continuum of common cancers, rare syndromes and mouse models. Nat. Rev. Cancer.

[57] Kopp K.L., Ralfkiaer U., Nielsen B.S., Gniadecki R., Woetmann A., Ødum N., Ralfkiaer E. (2013). Expression of miR-155 and miR-126 in situ in cutaneous T-cell lymphoma. APMIS.

[58] Moyal L., Barzilai A., Gorovitz B., Hirshberg A., Amariglio N., Jacob-Hirsch J., Maron L., Feinmesser M., Hodak E. (2013). miR-155 is involved in tumor progression of mycosis fungoides. Exp. Dermatol..

[59] Fredholm S., Willerslev-Olsen A., Met Ö., Kubat L., Gluud M., Mathiasen S.L., Friese C., Blümel E., Petersen D.L., Hu T. (2018). Special AT rich-binding1 protein (SATB1) in malignant T cells. J. Investig. Dermatol..

[60] Herrera A., Fredholm S., Cheng A., Mimitou E.P., Seffens A., Bar-Natan M., Sun A., Latkowski J.-A., Willerslew-Olsen A., Buus T.B. (2020). Low SATB1 Expression Promotes IL-5 and IL-9 Expression in Sézary Syndrome. J. Investig. Dermatol..

[61] Moyal L., Yehezkel S., Gorovitz B., Keren A., Gilhar A., Lubin I., Sherman S., Hodak E. (2017). Oncogenic role of microRNA-155 in mycosis fungoides: An in vitro and xenograft mouse model study. Br. J. Dermatol..

[62] Litvinov I.V., Cordeiro B., Fredholm S., Ødum N., Zargham H., Huang Y., Zhou Y., Pehr K., Kupper T.S., Woetmann A. (2014). Analysis of STAT4 expression in cutaneous T-cell lymphoma (CTCL) patients and patient-derived cell lines. Cell Cycle.

[63] Netchiporouk E., Litvinov I.V., Moreau L., Gilbert M., Sasseville D., Duvic M. (2014). Deregulation in STAT signaling is important for cutaneous T-cell lymphoma (CTCL) pathogenesis and cancer progression. Cell Cycle.

[64] Wu M., Duan Q., Liu X., Zhang P., Fu Y., Zhang Z., Liu L., Cheng J., Jiang H. (2020). MiR-155-5p promotes oral cancer progression by targeting chromatin remodeling gene ARID2. Biomed. Pharmacother..

[65] Seto A.G., Beatty X., Lynch J.M., Hermreck M., Tetzlaff M., Duvic M., Jackson A.L. (2018). Cobomarsen, an oligonucleotide inhibitor of miR-155, co-ordinately regulates multiple survival pathways to reduce cellular proliferation and survival in cutaneous T-cell lymphoma. Br. J. Haematol..

[66] Celik H., Koh W.K., Kramer A.C., Ostrander E.L., Mallaney C., Fisher D.A.C., Xiang J., Wilson W.C., Martens A., Kothari A. (2018). JARID2 Functions as a Tumor Suppressor in Myeloid Neoplasms by Repressing Self-Renewal in Hematopoietic Progenitor Cells. Cancer Cell.

[67] Su C.-L., Deng T.-R., Shang Z., Xiao Y. (2015). JARID2 inhibits leukemia cell proliferation by regulating CCND1 expression. Int. J. Hematol..

[68] Litvinov I.V., Netchiporouk E., Cordeiro B., Zargham H., Pehr K., Gilbert M., Zhou Y., Moreau L., Woetmann A., Ødum N. (2014). Ectopic expression of embryonic stem cell and other developmental genes in cutaneous T-cell lymphoma. Oncoimmunology.

[69] Nakagawa R., Leyland R., Meyer-Hermann M., Lu D., Turner M., Arbore G., Phan T.G., Brink R., Vigorito E. (2016). MicroRNA-155 controls affinity-based selection by protecting c-MYC+ B cells from apoptosis. J. Clin. Investig..

[70] Zargar S., Tomar V., Shyamsundar V., Vijayalakshmi R., Somasundaram K., Karunagaran D. (2019). A Feedback Loop between MicroRNA 155 (miR-155), Programmed Cell Death 4, and Activation Protein 1 Modulates the Expression of miR-155 and Tumorigenesis in Tongue Cancer. Mol. Cell. Biol..

[71] Espadinha A.-S., Prouzet-Mauléon V., Claverol S., Lagarde V., Bonneu M., Mahon F.-X., Cardinaud B. (2017). A tyrosine kinase-STAT5-miR21-PDCD4 regulatory axis in chronic and acute myeloid leukemia cells. Oncotarget.

[72] Cristofoletti C., Bresin A., Picozza M., Picchio M.C., Monzo F., Helmer Citterich M., Passarelli F., Frezzolini A., Scala E., Monopoli A. (2019). Blood and skin-derived Sezary cells: Differences in proliferation-index, activation of PI3K/AKT/mTORC1 pathway and its prognostic relevance. Leukemia.

[73] Zeng T., Zhang Q., Yu X., Gao X., Qiu Y. (2018). Inhibition of cell migration and invasion and promotion of cell apoptosis by overexpression of programmed cell death 4 (PDCD4) in cervical cancer Siha cells. Int. J. Clin. Exp. Pathol..

[74] Long J., Yin Y., Guo H., Li S., Sun Y., Zeng C., Zhu W. (2019). The mechanisms and clinical significance of PDCD4 in colorectal cancer. Gene.

[75] Kumar R., Selth L.A., Schulz R.B., Tay B.S., Neilsen P.M., Callen D.F. (2011). Genome-wide mapping of ZNF652 promoter binding sites in breast cancer cells. J. Cell. Biochem..

[76] Neilsen P.M., Noll J.E., Mattiske S., Bracken C.P., Gregory P.A., Schulz R.B., Lim S.P., Kumar R., Suetani R.J., Goodall G.J. (2013). Mutant p53 drives invasion in breast tumors through up-regulation of miR-155. Oncogene.

[77] Jiang D., Aguiar R.C.T. (2014). MicroRNA-155 controls RB phosphorylation in normal and malignant B lymphocytes via the noncanonical TGF-β1/SMAD5 signaling module. Blood.

[78] Rai D., Kim S.-W., McKeller M.R., Dahia P.L.M., Aguiar R.C.T. (2010). Targeting of SMAD5 links microRNA-155 to the TGF-beta pathway and lymphomagenesis. Proc. Natl. Acad. Sci. USA.

[79] Fuchs O., Simakova O., Klener P., Cmejlova J., Zivny J., Zavadil J., Stopka T. (2002). Inhibition of Smad5 in human hematopoietic progenitors blocks erythroid differentiation induced by BMP4. Blood Cells Mol. Dis..

[80] Zhang Y., Song J., Rutenberg D., Sokol L. (2020). Hepatic Infiltration with Malignant T-cells Manifesting as Impending Acute Liver Failure in Sezary Syndrome. Mediterr. J. Hematol. Infect. Dis..

[81] Jiang H., Cao H.-J., Ma N., Bao W.-D., Wang J.-J., Chen T.-W., Zhang E.-B., Yuan Y.-M., Ni Q.-Z., Zhang F.-K. (2020). Chromatin remodeling factor ARID2 suppresses hepatocellular carcinoma metastasis via DNMT1-Snail axis. Proc. Natl. Acad. Sci. USA.

[82] Benoit B.M., Jariwala N., O’Connor G., Oetjen L.K., Whelan T.M., Werth A., Troxel A.B., Sicard H., Zhu L., Miller C. (2017). CD164 identifies CD4+ T cells highly expressing genes associated with malignancy in Sézary syndrome: The Sézary signature genes, FCRL3, Tox, and miR-214. Arch. Dermatol. Res..

[83] Kohnken R., McNeil B., Wen J., McConnell K., Grinshpun L., Keiter A., Chen L., William B., Porcu P., Mishra A. (2019). Preclinical Targeting of MicroRNA-214 in Cutaneous T-Cell Lymphoma. J. Investig. Dermatol..

[84] Fang L.-L., Wang X.-H., Sun B.-F., Zhang X.-D., Zhu X.-H., Yu Z.-J., Luo H. (2017). Expression, regulation and mechanism of action of the miR-17-92 cluster in tumor cells (Review). Int. J. Mol. Med..

[85] Moyal L., Gorovitz-Haris B., Yehezkel S., Jacob-Hirsch J., Bershtein V., Barzilai A., Rotem C., Sherman S., Amitay-Laish I., Feinmesser M. (2019). Unilesional mycosis fungoides is associated with increased expression of microRNA-17~92 and T helper 1 skewing. Br. J. Dermatol..

[86] Hanahan D., Weinberg R.A. (2000). The Hallmarks of Cancer. Cell.

[87] Xia L., Wu L., Xia H., Bao J., Li Q., Chen X., Xia R. (2019). miR-337 suppresses cutaneous T-cell lymphoma via the STAT3 pathway. Cell Cycle.

[88] Ito M., Teshima K., Ikeda S., Kitadate A., Watanabe A., Nara M., Yamashita J., Ohshima K., Sawada K., Tagawa H. (2014). MicroRNA-150 inhibits tumor invasion and metastasis by targeting the chemokine receptor CCR6, in advanced cutaneous T-cell lymphoma. Blood.

[89] Kitadate A., Ikeda S., Teshima K., Ito M., Toyota I., Hasunuma N., Takahashi N., Miyagaki T., Sugaya M., Tagawa H. (2016). MicroRNA-16 mediates the regulation of a senescence-apoptosis switch in cutaneous T-cell and other non-Hodgkin lymphomas. Oncogene.

[90] Gupta S., Silveira D.A., Barbé-Tuana F.M., Mombach J.C.M. (2020). Integrative data modeling from lung and lymphatic cancer predicts functional roles for miR-34a and miR-16 in cell fate regulation. Sci. Rep..

[91] Wei H., Liu R., Guo X., Zhou Y., Sun B., Wang J. (2019). miRNA-135a regulates Hut78 cell proliferation via the GATA-3/TOX signaling pathway. Mol. Med. Rep..

[92] McGirt L.Y., Adams C.M., Baerenwald D.A., Zwerner J.P., Zic J.A., Eischen C.M. (2014). miR-223 regulates cell growth and targets proto-oncogenes in mycosis fungoides/cutaneous T-cell lymphoma. J. Investig. Dermatol..

[93] Ohmatsu H., Sugaya M., Suga H., Morimura S., Miyagaki T., Kai H., Kagami S., Fujita H., Asano Y., Tada Y. (2012). Serum IL-31 levels are increased in patients with cutaneous T-cell lymphoma. Acta Derm. Venereol..

[94] Möbs M., Gryzik S., Haidar A., Humme D., Beyer M., Vandersee S. (2015). Analysis of the IL-31 pathway in Mycosis fungoides and Sézary syndrome. Arch. Dermatol. Res..

[95] Malek M., Gleń J., Rębała K., Kowalczyk A., Sobjanek M., Nowicki R., Ruckemann-Dziurdzińska K., Sokołowska-Wojdyło M. (2015). Il-31 does not correlate to pruritus related to early stage cutaneous T-cell lymphomas but is involved in pathogenesis of the disease. Acta Derm. Venereol..

[96] Han Q., Liu D., Convertino M., Wang Z., Jiang C., Kim Y.H., Luo X., Zhang X., Nackley A., Dokholyan N.V. (2018). miRNA-711 binds and activates TRPA1 extracellularly to evoke acute and chronic pruritus. Neuron.

[97] Pluta L., Yousefi B., Damania B., Khan A.A. (2019). Endosomal TLR-8 Senses microRNA-1294 Resulting in the Production of NFḱB Dependent Cytokines. Front. Immunol..

[98] Bayraktar R., Bertilaccio M.T.S., Calin G.A. (2019). The Interaction between Two Worlds: MicroRNAs and Toll-Like Receptors. Front. Immunol..

[99] Zhang Z.-J., Guo J.-S., Li S.-S., Wu X.-B., Cao D.-L., Jiang B.-C., Jing P.-B., Bai X.-Q., Li C.-H., Wu Z.-H. (2018). TLR8 and its endogenous ligand miR-21 contribute to neuropathic pain in murine DRG. J. Exp. Med..

[100] Hanna J., Hossain G.S., Kocerha J. (2019). The Potential for microRNA Therapeutics and Clinical Research. Front. Genet..

[101] Ralfkiaer U., Hagedorn P.H., Bangsgaard N., Løvendorf M.B., Ahler C.B., Svensson L., Kopp K.L., Vennegaard M.T., Lauenborg B., Zibert J.R. (2011). Diagnostic microRNA profiling in cutaneous T-cell lymphoma (CTCL). Blood.

[102] Marstrand T., Ahler C.B., Ralfkiaer U., Clemmensen A., Kopp K.L., Sibbesen N.A., Krejsgaard T., Litman T., Wasik M.A., Bonefeld C.M. (2014). Validation of a diagnostic microRNA classifier in cutaneous T-cell lymphomas. Leuk. Lymphoma.

[103] Manso R., Martínez-Magunacelaya N., Eraña-Tomás I., Monsalvez V., Rodríguez-Peralto J.L., Ortiz-Romero P.-L., Santonja C., Cristóbal I., Piris M.A., Rodríguez-Pinilla S.M. (2018). Mycosis fungoides progression could be regulated by microRNAs. PLoS ONE.

[104] Dusílková N., Bašová P., Polívka J., Kodet O., Kulvait V., Pešta M., Trněný M., Stopka T. (2017). Plasma miR-155, miR-203, and miR-205 are Biomarkers for Monitoring of Primary Cutaneous T-Cell Lymphomas. Int. J. Mol. Sci..

[105] McGirt L., Baerenwald D., Vonderheid E., Eischen C. (2015). Early changes in miRNA expression are predictive of response to extracorporeal photopheresis in cutaneous T-cell lymphoma. J. Eur. Acad. Derm. Venereol..

[106] Manfè V., Biskup E., Willumsgaard A., Skov A.G., Palmieri D., Gasparini P., Laganá A., Woetmann A., Ødum N., Croce C.M. (2013). cMyc/miR-125b-5p signalling determines sensitivity to bortezomib in preclinical model of cutaneous T-cell lymphomas. PLoS ONE.

[107] Manfè V., Biskup E., Rosbjerg A., Kamstrup M., Skov A.G., Lerche C.M., Lauenborg B.T., Odum N., Gniadecki R. (2012). miR-122 regulates p53/Akt signalling and the chemotherapy-induced apoptosis in cutaneous T-cell lymphoma. PLoS ONE.

[108] Abe F., Kitadate A., Ikeda S., Yamashita J., Nakanishi H., Takahashi N., Asaka C., Teshima K., Miyagaki T., Sugaya M. (2017). Histone deacetylase inhibitors inhibit metastasis by restoring a tumor suppressive microRNA-150 in advanced cutaneous T-cell lymphoma. Oncotarget.

[109] Krejsgaard T., Lindahl L.M., Mongan N.P., Wasik M.A., Litvinov I.V., Iversen L., Langhoff E., Woetmann A., Odum N. (2017). Malignant inflammation in cutaneous T-cell lymphoma—A hostile takeover. Semin. Immunopathol..

[110] Mishra A., La Perle K., Kwiatkowski S., Sullivan L.A., Sams G.H., Johns J., Curphey D.P., Wen J., McConnell K., Qi J. (2016). Mechanism, Consequences, and Therapeutic Targeting of Abnormal IL15 Signaling in Cutaneous T-cell Lymphoma. Cancer Discov..

[111] Kohnken R., Wen J., Mundy-Bosse B., McConnell K., Keiter A., Grinshpun L., Hartlage A., Yano M., McNeil B., Chakravarti N. (2018). Diminished microRNA-29b level is associated with BRD4-mediated activation of oncogenes in cutaneous T-cell lymphoma. Blood.

[112] Querfeld C., Foss F.M., Kim Y.H., Pinter-Brown L., William B.M., Porcu P., Pacheco T., Haverkos B.M., DeSimone J., Guitart J. (2018). Phase 1 Trial of Cobomarsen, an Inhibitor of Mir-155, in Cutaneous T Cell Lymphoma. Blood.

[113] Querfeld C., Foss F.M., Pinter-Brown L.C., Porcu P., William B.M., Pacheco T., Haverkos B.M., Kim Y.H., Guitart J., Halwani A.S. (2017). Phase 1 Study of the Safety and Efficacy of MRG-106, a Synthetic Inhibitor of microRNA-155, in CTCL Patients. Blood.

[114] Nielsen M., Nissen M.H., Gerwien J., Zocca M.-B., Rasmussen H.M., Nakajima K., Röpke C., Geisler C., Kaltoft K., Ødum N. (2002). Spontaneous interleukin-5 production in cutaneous T-cell lymphoma lines is mediated by constitutively activated Stat3. Blood.

[115] Verma N.K., Davies A.M., Long A., Kelleher D., Volkov Y. (2010). STAT3 knockdown by siRNA induces apoptosis in human cutaneous T-cell lymphoma line Hut78 via downregulation of Bcl-xL. Cell. Mol. Biol. Lett..

[116] Cremer T.J., Fatehchand K., Shah P., Gillette D., Patel H., Marsh R.L., Besecker B.Y., Rajaram M.V.S., Cormet-Boyaka E., Kanneganti T.-D. (2012). MiR-155 induction by microbes/microbial ligands requires NF-κB-dependent de novo protein synthesis. Front. Cell Infect. Microbiol..

